# *Aedes albopictus* abundance and phenology along an altitudinal gradient in Lazio region (central Italy)

**DOI:** 10.1186/s13071-022-05215-9

**Published:** 2022-03-18

**Authors:** Federico Romiti, Riccardo Casini, Adele Magliano, Arianna Ermenegildi, Claudio De Liberato

**Affiliations:** Istituto Zooprofilattico Sperimentale del Lazio e Della Toscana ‘M. Aleandri’, Via Appia Nuova 1411, 00178 Rome, Italy

**Keywords:** Invasive mosquitoes, Distribution, Culicidae, Disease vector, Risk map

## Abstract

**Background:**

The Asian tiger mosquito *Aedes albopictus* (Skuse 1894), which is native to Southeast Asia, is among the top 100 invasive species worldwide and one of the most troubling vector species. It has become established in more than 20 European countries. Since its arrival in Italy in the 1990s, the species has colonized all the regions of the country, up to an altitude of 600 m. Nevertheless, no thorough investigation has ever been performed to confirm or extend its elevation limit (EL) in Italy.

**Methods:**

To define the EL of *Ae. albopictus* and analyse its phenology along an altitudinal gradient, we carried out an investigation by means of ovitraps placed in Lazio region, central Italy. Sampling was performed on a weekly basis in 13 villages within five 200-m altitudinal ranges [0–1000 m above sea level (asl)], with the addition of higher localities to the species range whenever the species was recorded in the highest range.

**Results:**

*Aedes albopictus* has colonized sites well beyond its known EL, with established populations at 900 m asl and positive ovitraps recorded at 1193 m asl. The relationship between egg abundance and elevation was described by an exponential decay regression, which predicted an EL for oviposition at 1015 m asl. In the active season, egg-laying started earlier at low altitude and ended earlier within the highest altitudinal range. *Aedes albopictus* abundance and activity period (number of days active) decreased, respectively, by 95% and 34% from the lowest to the highest altitudinal range.

**Conclusions:**

Using data from the present study, the altitudinal limit of *Ae. albopictus* in central Italy was updated from 600 to 900 m asl. In addition, established populations were predicted to exist up to 1015 m asl. Considering that up to 99.5% of Lazio region’s inhabitants could potentially be affected by *Aedes*-borne virus outbreaks, the surveillance area for *Ae. albopictus* should be expanded accordingly. However, our results also indicate that *Ae. albopictus* surveillance programs need to be revised in order to harmonize the resources earmarked for these with the altitudinal changes in the phenology of this species.

**Graphical abstract:**

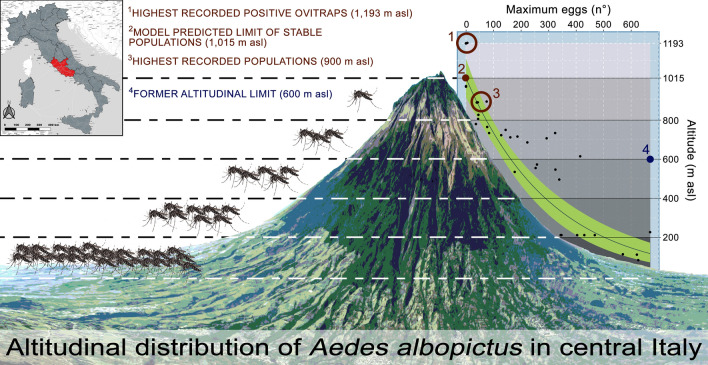

## Background

The Asian tiger mosquito *Aedes albopictus* (Skuse 1894), which is native to Southeast Asia, is one of the most important vectors affecting human health worldwide, ranking second, among the invasive mosquitoes of the genus *Aedes*, only to the yellow fever mosquito *Aedes aegypti* [1, 2]. The species is known to experimentally transmit more than 26 arboviruses, and has been involved in the transmission of dengue and chikungunya viruses during recent outbreaks [3–6]. *Aedes albopictus* is one of the top 100 invasive species and has successfully colonized tropical and temperate regions worldwide; it arrived in Europe in the 1970s, and is now present in more than 20 countries of the European Union [7–10]. The species was introduced into Italy in 1990 through the importation of tyres, and is nowadays widespread and commonly found in every region of the country [11, 12]. Despite the fact that *Ae. albopictus* is a zoophilic tree-hole breeder in its native range, its strong physiological and ecological plasticity allow it to feed on alternative food sources such as domestic animals and humans, and to thrive in human-made habitats [13–16]. Indeed, this great adaptability has allowed the species to colonize large urban areas as well as tiny villages in southern Europe [17–19]. Climate change is expected to favour the range expansion of *Ae. albopictus*, shifting its distribution limits polewards and to higher altitudes [20, 21]. Particularly, increasing winter and summer temperatures, together with changes in precipitation patterns, might reduce its developmental and diapause time, increase adult survival, and lead to an increase in the provision of water to potential breeding sites [22–26].

The global expansion of the range of *Ae. albopictus*, which is driven by human movement and commercial activities, is frequently followed by the spread of *Aedes*-borne viral diseases [27, 28]. This poses a serious risk to health according to the World Health Organization [2]. The European Centre for Disease Prevention and Control, as a consequnce of the spread of *Aedes* spp. in Europe, has stressed the need for a comprehensive understanding of the risks associated with mosquito vectors and the importance of implementing surveillance plans [29, 30]. In support of these aims, following the chikungunya outbreaks that occurred in Italy in 2007 and 2017 [31, 32], the Italian Ministry of Health issued a national plan for the surveillance and response to arboviruses (PNA) transmitted by invasive mosquitoes (*Aedes* spp.) [33, 34]. In Italy, the country in which the tiger mosquito has reached its greatest European distribution [35], the species is expected to spread in terms of altitude, similarly to what has occurred in Albania, where populations were recently found at above 1000 m above sea level (asl) [18]. In this respect, as hills and mountains represent 76.83% of the land surface of Italy, health threats associated with *Ae. albopictus* may even increase, placing more people at risk of viral diseases transmitted by this vector [36]. Despite the importance of investigating the elevation limit (EL) of *Ae. albopictus* in Italy, both from an ecological and a sanitary point of view, a thorough study of this has never been performed in the country. In fact, to date, based on old data, the above-mentioned PNA limits the extent of *Ae. albopictus* surveillance to areas below 600 m asl [12]. The urgent need to overcome this lack of data, as highlighted by recent dengue and chikungunya outbreaks in Italy [6, 32], encouraged us to undertake this study. To achieve the aims of this study, we carried out an investigation on *Ae. albopictus* in central Italy along an altitudinal gradient to define its upper distribution limit and analyse changes in its abundance and oviposition activity.

## Methods

### Study area

The study was carried out in Lazio region, central Italy, which is characterized by a warm temperate climate according to the Köppen-Geiger classification [37]. The sampling localities were selected along two transects that followed an altitudinal gradient from the lowlands to the highest inhabited centres of the region (towns or small villages). Sites were chosen where no insecticide treatments were scheduled by the local authorities.

**Fig. 1:**
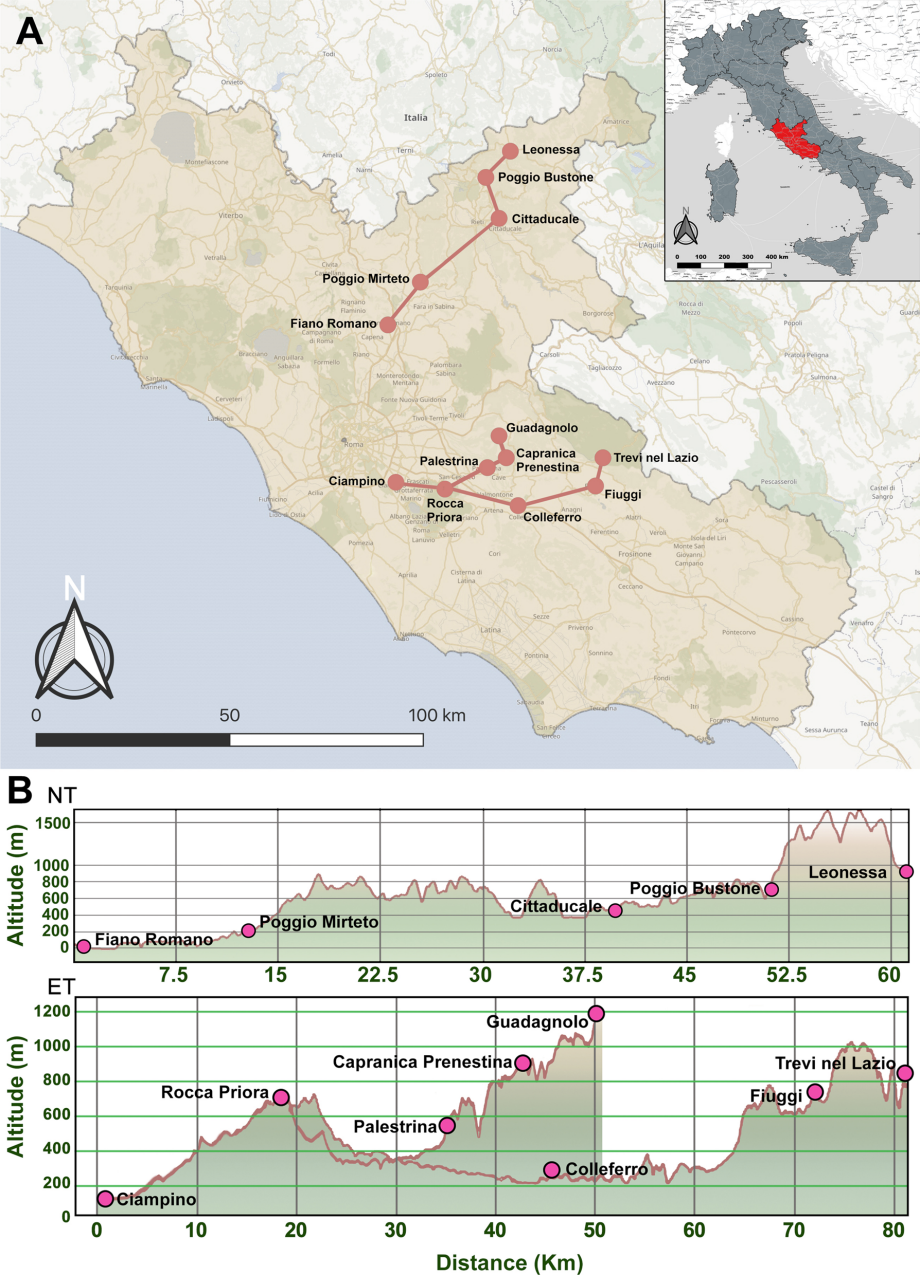
Map of the study area in Lazio region, central Italy, with sampling localities (dots) and transects (lines) highlighted (**a**). Elevation profile of each transect produced using the Profile Tool in QGIS from the digital elevation model of Italy, to visualize the sectional view as it would appear when connecting one locality to the next by a straight line (**b**).* NT* North transect,* ET* east transect

The first transect extended from southwest to northeast (NT), and the second one in an easterly direction from Rome urban area (ET) (Fig. 1a; Table 1). Each transect was divided into five 200-m altitudinal ranges, from 0 to 1000 m asl, as follows: 0–200, 201–400, 401–600, 601–800, 801–1000 m. In each range, at least one sampling locality was selected. At the beginning of the study, the initial set of localities consisted of five inhabited centres along NT, with a maximum altitude of 980 m asl (Leonessa municipality), and six along ET, where the maximum altitude was 830 m asl (Trevi nel Lazio municipality) (Fig. 1b). Given the known anthropophily of the species, and taking into account its known altitudinal limit in Italy [12, 38], localities above 600 m altitude were chosen following a criterion based on human population count. For this purpose, raster data based on population counts (30 arc-second resolution), consistent with national censuses for the year 2020, were obtained from the National Aeronautics and Space Administration’s Earth Observing System Data and Information System [39]. Considering the reduction in human population density at increasing altitude [40], the mean population count of the highest locality (Leonessa) was considered the minimum population threshold for locality/trap site selection. A circular buffer (diameter 400 m) was drawn in QGIS to calculate the mean population count around each trap in Leonessa; the procedure was then repeated for each locality above 600 m asl to select trap sites where population count was not below the calculated threshold. As the sampling progressed, whenever the occurrence of *Ae. albopictus* was detected at a higher locality, a further inhabited centre, at a higher altitude, was added to the transect. This procedure, through the addition of Capranica Prenestina (~ 890 m) and Guadagnolo (~ 1190 m), led to eight localities for ET, which gave 13 localities in total. Due to their low population density, the population criterion was ignored for these late additions to the transect.

**Table 1:** Summarized information on sampling localities

Transect	Locality	Mean OT altitude (m asl)	Coordinates
NT	Fiano Romano	98	42°10′N, 12°36′E
	Poggio Mirteto	238	42°16′N, 12°41′E
	Cittaducale	517	42°23′N, 12°57′E
	Poggio Bustone	750	42°30′N, 12°53′E
	Leonessa	972	42°34′N, 12°57′E
ET	Ciampino	125	41°48′N, 12°36′E
	Colleferro	212	41°43′N, 13°00′E
	Palestrina	558	41°50′N, 12°54′E
	Fiuggi	686	41°48′N, 13°13′E
	Rocca Priora	717	41°47′N, 12°46′E
	Trevi nel Lazio	810	41°51′N, 13°15′E
	Capranica Prenestina	892	41°52′N, 12°57′E
	Guadagnolo	1193	41°54′N, 12°55′E

* NT* North transect,* ET* east transect, *OT* ovitrap,* asl* above sea level

### Sampling design

Sampling was carried out over a 6-month period (end of May through November 2021) by means of standard ovitraps (OTs) consisting of 400-ml black plastic containers three-quarters filled with tap water and equipped with a Masonite strip (15 × 3 cm) for oviposition. At least three OTs were placed in each locality at ground level, in sheltered and shaded places, and were left in the same position throughout the whole study period. OT sites were selected according to the presence, in the surrounding area, of at least two of the following: vegetation (house or urban garden), gathering place (e.g. café, train or bus station), or water-collecting containers (e.g. saucers or drinking fountains). The Masonite strips were collected weekly and eggs were counted under a stereomicroscope. To confirm *Aedes albopictus* identification and rule out the presence of other invasive *Aedes* species reported for Italy (i.e. *Aedes koreicus* and *Aedes japonicus*), all Masonite strips from the highest localities (801–1000 m asl), and randomly chosen strips from lower localities, were placed into water with kitten chow to allow eggs to hatch and larvae to develop up to the adult stage [41]. Adult mosquitoes were identified using morphological identification keys [42, 43].

### Statistical analysis

#### Altitudinal limit

To define the altitudinal limit of *Ae. albopictus*, identify any municipalities free of this species, and outline its changes in abundance with increasing elevation, the relationship between OT altitude and maximum number of eggs laid weekly (MEggs) was investigated. To determine the elevation threshold where MEggs dropped to zero, four models were performed: linear and segmented regression, exponential decay and generalized additive model (GAM). The GAM model was fitted using a restricted maximum likelihood method and goodness-of-fit of the model was checked using the gam.check function in the mgcv R package, to choose the number of basis functions (*k*) [44–46]. The best-fitting model was selected on the basis of the second-order Akaike information criterion (AICc) value and the ΔAICc between models [47, 48]; then the intercept was calculated to highlight the predicted EL where MEggs drops to zero. Minimum and maximum values of MEggs were calculated for each altitudinal range, according to the best-fitting model formula. The upper limit of the highest range (801–1000 m asl) was extended to the predicted EL and, above that, a further range was added to include areas with predicted MEggs equal to zero. To visualize the results and provide a useful tool for managing surveillance activities in Lazio region, a map reporting altitudinal ranges and predicted minimum and maximum MEggs values was produced. The mean altitude of each municipality was calculated using QGIS software [49] and elevation data from a digital elevation model of Italy (10-m resolution) [50].

#### Phenological metrics

Phenological metrics were calculated for each altitudinal range by applying the novel method proposed by Edwards and Crone [51]. This method was developed to compare phenologies of different years from count data collected at the same site. Here we compared phenological patterns of different altitudinal ranges by calculating the weekly mean number of eggs laid (mEggs_w_) for each range during the whole sampling period, namely from weeks 23 to 49. The localities considered in this analysis (*n* = 11) were grouped into five 200-m altitudinal ranges (0–200, 201–400, 401–600, 601–800, 801–1000 m); two localities were excluded because the non-zero survey data were insufficient to fit a Gaussian model (see “General outcomes”). Gaussian models were fitted using generalized linear models with a negative binomial and Poisson distribution, both with the log link function. mEggs_w_ was the dependent variable and day of year (DOY) and DOY^2^ were independent variables. We preferred to use mEggs_w_ rather than the daily mean of eggs laid because *Ae. albopictus* surveillance activities are carried out on a weekly basis in accordance with the PNA [34]. We used DOY rather than week number to ease the interpretation of model results and visualization. To account for the interaction between DOY and altitudinal range, namely to fit each range separately, range was added as a categorical factor in the model formulae. The best-fitting model was selected as described in the “Altitudinal limit” section. Then, starting from the coefficients of the intercept, the linear and quadratic terms of the best-fitting Gaussian model, we calculated the phenological metrics for each altitudinal range, following the procedure of Edwards and Crone [51]. The main phenological metrics were as follows: DOY when the peak of oviposition occurred (*µ*), SD for the Gaussian distribution (*σ*), index of egg abundance (*N*) and estimated peak of mEggs_w_ (*h*). For these metrics, 95% confidence intervals and SE were calculated using, respectively, parametric bootstrapping (MASS R package [52]) and the delta method (msm R package [53, 54]). The time interval within which 80% of the eggs (mEggs_w_) were laid (80EA) was calculated as the number of days between the 10th and 90th percentiles. The observable activity period was calculated as the number of days when the predicted mEggs_w_ was > 1 [55]. Furthermore, the first and last DOY of egg-laying activity were determined, respectively, as the day when mEggs_w_ exceeded zero or dropped to zero, according to the predicted values from the Gaussian models. To test for asymmetry in the phenological patterns of egg-laying activity among the altitudinal ranges, a GAM model with a cubic regression spline was fitted, with the Gaussian model’s residuals as the dependent variable and DOY as independent variables. The effective* df* was checked to highlight any significant deviation from a straight line, i.e. the presence of asymmetry in the phenological patterns [44, 51].

## Results

### General outcomes

A total of 1045 Masonite strips were examined during the study period, of which 50.7% were positive (i.e. at least one egg was present). *Aedes albopictus* was found in all but one of the sampled localities (*n* = 12), the only exception being Leonessa, the highest locality on NT (mean altitude of OTs = 972 m asl). The species was present in all altitudinal ranges, from the lowest locality (Fiano Romano, NT; mean OT elevation = 98 m asl) to the highest (Guadagnolo, ET; mean OT elevation = 1193 m asl). Notably, in Guadagnolo only two OTs were positive, with four and five eggs, respectively, during two consecutive surveys (20 and 27 September). The number of eggs laid weekly exceeded 600 five times, in two OTs within the lowest altitudinal range (0–200 m asl), and in one OT at 201–400 m asl, between 19 July and 4 October. All the identified adult mosquitoes were *Ae. albopictus*.

### Altitudinal limit

**Fig. 2:**
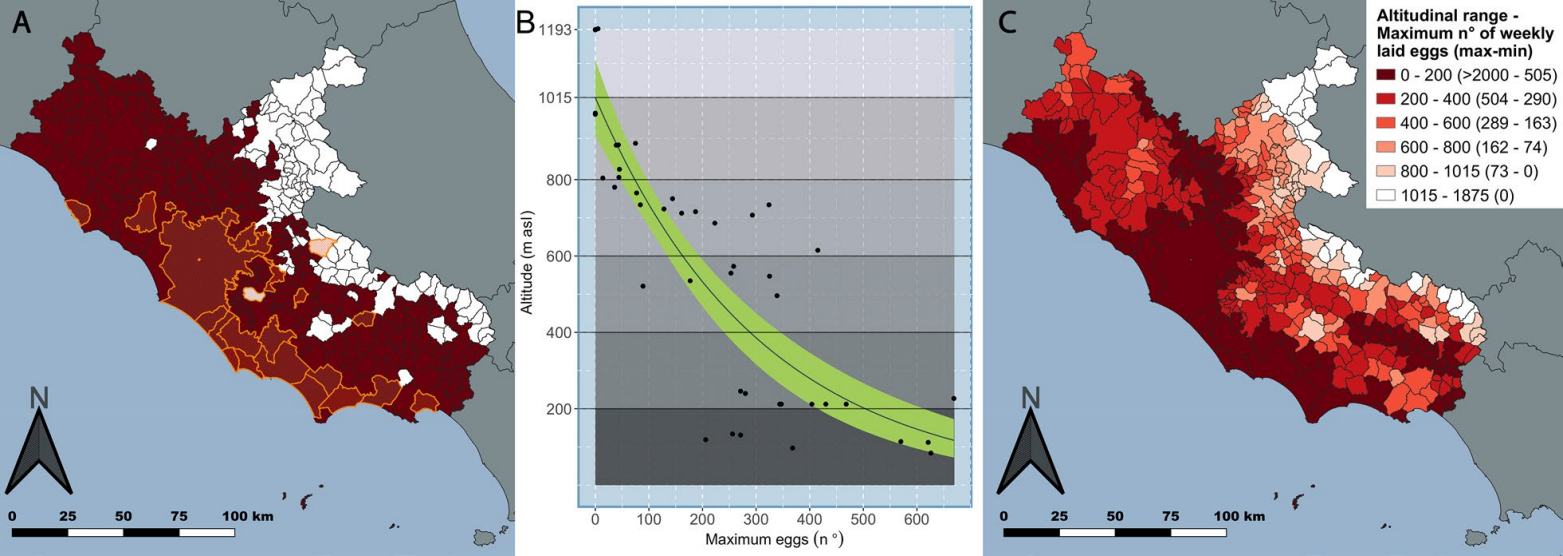
**a** Map of Lazio region showing the municipalities in red (dark grey in the monochrome version) (*n* = 281) where *Aedes albopictus* surveillance should be carried out according to the national plan for the surveillance and response to arboviruses (PNA), namely those municipalities with a mean altitude < 600 m above sea level (asl). The boundaries of municipalities with on-going surveillance (*n* = 24) have been highlighted in orange (light grey in the monochrome version). The remaining municipalities with a mean altitude above 600 m asl (*n* = 97) are in white. **b** Relationship between the weekly maximum (*max*) number of eggs laid (*MEggs*) recorded in each ovitrap (OT) and altitude range, as described by the best-fitting model (exponential decay). Bias-corrected and accelerated confidence intervals (CIs) calculated with 1000 bootstrap replicates are in light green (white in the monochrome version). The grey shaded areas indicate the decreasing abundance of MEggs at increasing altitude till the highest locality where the species was recorded.** c** Altitudinal and MEggs ranges (in parentheses) according to the exponential decay results, with municipalities in decreasingly intense shades of red (grey in the monochrome version) at increasing altitude (with decreasing MEggs abundance). *max* Maximum, * min* Minimum

The model comparison indicated that the relationship between MEggs and altitude was best described by an exponential decay regression. GAM, segmented and linear regressions were discarded considering their |ΔAICc| with respect to the first ranked model (Table 2). The estimated intercept from the exponential decay indicated that MEggs dropped to zero above 1000 m asl, as shown by the model trend (Fig. 2b). Two maps were produced for Lazio region to compare the municipalities where *Ae. albopictus* surveillance should be carried out according to the PNA (under 600 m asl), where it is actually performed (Fig. 2a), and the predicted occurrence and relative abundance of the species according to the relationship between MEggs and altitude (Fig. 2c). According to the predicted EL, in Lazio region, *Ae. albopictus* might be present in 354 municipalities, although at different abundances, with a geographical distribution of 15,617.18 km^2^ (~ 91% of Lazio region), and 99.5% of the inhabitants of this region may exposed to it [56], with only 24 municipalities located above the species EL (comprising an area of 1589.23 km^2^, which is ~ 9% of the total area of the region) (Fig. 2c).

**Table 2:** Summary of the results for the exponential decay regression, generalized additive model (*GAM*), and segmented and linear regression models, ordered according to their second-order Akaike information criterion (*AICc*) score and the absolute value of ΔAICc

Model	Formula	*df* (e*df*)	AICc	|ΔAICc|	Estimated intercept (± SE)	Intercept* P*-value	Adj. *R*^2^
Exponential decay	Altitude ~ 1015.00 × *e*^(−0.003 × MEggs)^	42	583.48	0	1015.00 (± 52.27)	< 2e−16	NA
GAM	Altitude ~ s(MEggs,* k* = 4, sp = 0.1)	2.22·	587.16	3.68	987.65 (± 26.86)	< 2e−16	0.72
Segmented regression	segmented.lm[lm, seg.Z = ~ MEggs, control = seg.control (n.boot = 10)]	40	588.26	4.78	1010.13 (± 55.36)	< 2e−16	0.72
Linear model (lm)	Altitude ~ − 1.46 × MEggs + 904.29	42	593.75	10.27	904.29 (± 46.33)	< 2e−16	0.66

The predicted values of altitude where the weekly maximum number of eggs laid (*MEggs*) by *Aedes albopictus* dropped to zero are reported as an estimated intercept. The GAM function was set with four basis functions (k = 4); the smooth function (s) was used to imply a flexible relation between *Altitude* and *MEggs* and the smoothing parameter (sp) was set to 0.1. The results of the gam.check function indicated that the number of basis functions was appropriate since *P*-value of GAM was 0.36 and *k*-index was close to 1 (0.97), with *k*’ = 3 [46]. The effective *df* (*edf*) of the GAM model indicated that the relation between *Altitude* and *MEggs* was similar to a quadratic curve (namely where *edf* = 2). The Estimated break-point (± SE) from segmented regression was 206 (± 56.29) and the slopes (± SE) of the two resulting linear regressions were − 2.66 (± 0.57) and − 0.76 (± 0.29)

*e* Euler's number (~ 2.71828);* seg.Z* the continuous covariate (*MEggs* in this formula), which is understood to have a piecewise-linear relationship with response (*Altitude*); *n.boot* number of bootstrap samples used in the bootstrap restarting algorithm; *Adj*. adjusted

### Phenological metrics

**Fig. 3:**
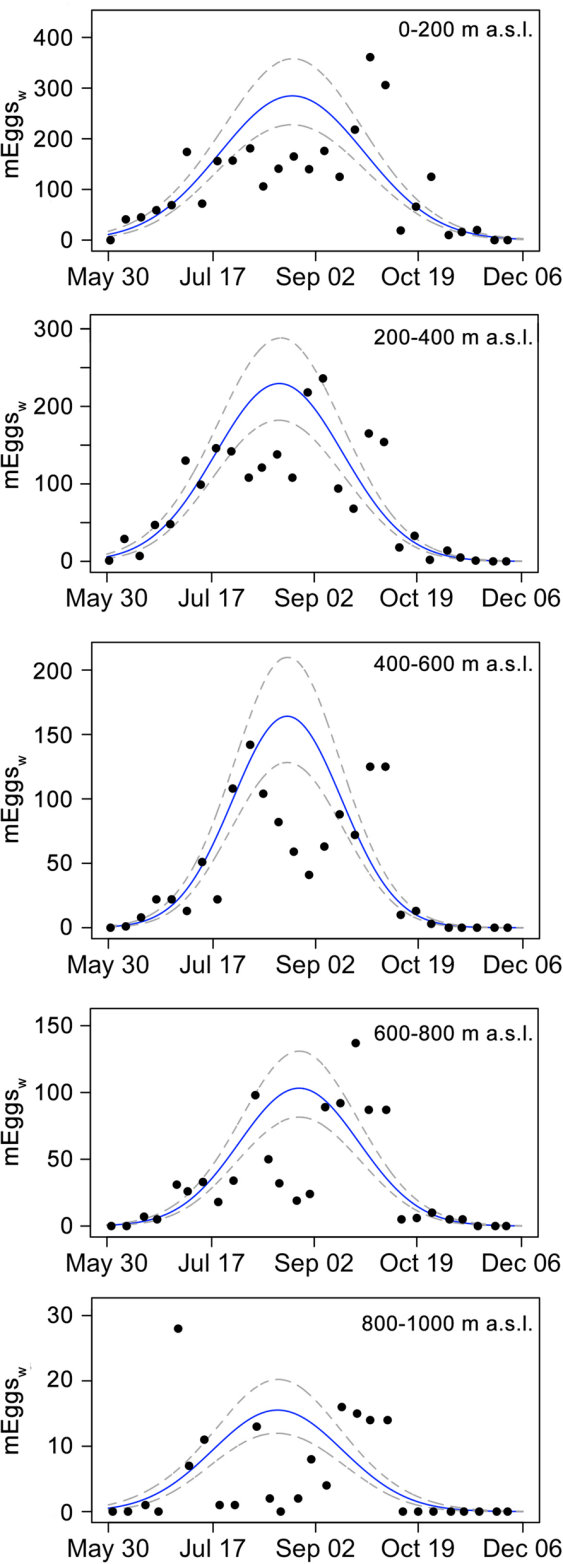
Gaussian model fitted to the seasonal trend of *Aedes albopictus* mean weekly number of eggs laid (*mEggs*_*w*_) at different altitudinal ranges. Blue solid lines (black in the monochrome version) show the best-fitting Gaussian model (negative binomial), dashed lines indicate the CIs calculated with the parametric bootstrapping approach. Note the change in scale of the* y*-axis (0–30 mEggs_w_ at the highest altitudinal range, and 0–400 mEggs_w_ at the lowest altitudinal range)

There was a higher deviance for the Gaussian model with the Poisson distribution compared to the one with a negative binomial (Gaussian_Poisson_—null deviance = 54,509.4 with 135* df*, residual deviance = 2420.5 with 120* df*; Gaussian_negative_binomial_—null deviance = 12,940.24 with 135* df*, residual deviance = 149.69 with 120* df*). The Gaussian model with a negative binomial distribution showed a higher goodness of fit when comparing the AICc (Gaussian_Poisson_ = 3021.81, Gaussian_negative_binomial_ = 1093.7, with |ΔAICc|= 1928.1). The phenological metrics reported in Table 3 were then calculated for each altitudinal range using the coefficients from the Gaussian_negative_binomial_ model. Peaks of oviposition activity occurred during the second half of August in each altitudinal range, with a mean SD of egg-laying activity (*σ*) of 29 days. Egg abundance index decreased by one order of magnitude from the lowest altitudinal range (0–200 m asl) to the highest, along with the predicted maximum value of mEggs_w_, as shown by the phenological patterns in Fig. 3. As expected, given the correlation between the observable activity period and egg abundance [55], the number of the days when predicted mEggs_w_ was > 1 decreased with increasing altitude, from ~ 220 days within the lowest range (0–200 m asl) to ~ 140 days within the highest range (801–1000 m asl). The time interval of 80EA, which had a mean of 74 days (± 3.5), did not show a clear decreasing trend with increasing altitude, since it is not correlated with egg abundance [57]. Higher egg abundance in the lower altitudinal ranges, which elongated the tails of the Gaussian distribution, gave an early predicted onset of oviposition activity at low altitude (end of April for 0–200 m asl) and an earlier end within the highest altitudinal range (early in November). Indeed, the actual recorded first and last positive OTs in the different altitudinal ranges were observed at DOY 158–323 (0–200 m), 151–320 (201–400 m), 159–299 (401–600 m), 167–313 (601–800 m) and 182–285 (801–1000 m). The GAM model indicated asymmetry in the phenological patterns among the altitudinal ranges. Indeed, the effective* df* of the smoothing term were 10.9 (*P*-value < 0.01) and the GAM check results indicated that the number of basis functions needed to describe the relation between the Gaussian model’s residuals and DOY was higher than 1 (*k*′ = 11, *k*-index = 0.87, *P*-value = 0.06).

**Table 3:** Summary of the results of the Gaussian models reporting the phenological metrics of egg-laying activity of *Aedes albopictus* for each altitudinal range

Altitudinal range	Localities	DOY peak (date in 2021)		SD of activity		Egg abundance index		Peak abundance^a^		80EA (no. of days)	Observable activity period^b^	First DOY^c^	Last DOY^d^
		*μ*	95% CI pb (SE dm)	*σ*	95% CI pb (SE dm)	*N*	95% CI pb (SE dm)	*h*	95% CI pb (SE dm)				
0–200 m asl	FIA, CIA	Day 234 (22 August)	227–241 (±3)	33.13	29.55–38.25 (±2.18)	23,643.80	16,868.39–34,549.23 (±4368.08)	285	185–448 (±65)	85	223	117	352
201–400 m asl	POM, COL	Day 229 (17 August)	223–234 (±3)	29.24	26.38–33.28 (±1.71)	16,815.62	11,603.87–24,807.18 (±3265.17)	229	146–364 (±53)	75	193	127	331
401–600 m asl	PAL, CIT	Day 232 (20 August)	227–237 (±2)	24.96	22.62–28.12 (±1.37)	10,268.70	6942.07–15,723.55 (±2145.98)	164	102–268 (±40)	64	159	148	316
601–800 m asl	POB, ROP, FIU	Day 238 (26 August)	232–244 (±3)	27.45	24.74–31.38 (±1.63)	7101.87	4873.04–10,560.85 (±1429.89)	103	65–164 (±25)	70	167	149	327
801–1000 m asl	TRE, CAP	Day 228 (16 August)	218–236 (±4)	29.71	25.17–37.53 (±2.88)	1156.30	788.46–1788.27 (±243.17)	16	9–27 (±4)	76	139	150	305

The main phenological metrics [day of year (*DOY*) when the peak of oviposition occurred (*µ*), SD for the Gaussian distribution (*σ*), index of egg abundance (*N*), and estimated peak of mean number of eggs laid weekly (*h*)] are reported with their 95% confidence intervals (*CI*) and SEs. The parametric bootstrapping approach (*pb*) used the variance–covariance matrix of the coefficient estimates from the fitted Gaussian models with 10,000 simulated coefficients. The symmetrical SE was calculated according to the delta method (*dm*)

* FIA* Fiano Romano, *CIA* Ciampino, *POM* Poggio Mirteto, *COL* Colleferro,* PAL* Palestrina,* CIT* Cittaducale,* POB* Poggio Bustone,* ROP* Rocca Priora,* FIU* Fiuggi ,*TRE* Trevi nel Lazio, *CAP* Capranica Prenestina, *80EA* 80% of egg-laying activity

^a^Peak abundance is the predicted maximum value of mEggs_w_.

^b^Observable activity period is the number of days when the predicted mEggs_w_ is > 1

^c^First DOY is the predicted day of egg-laying onset

^d^Last DOY is the predicted day when egg-laying stopped

## Discussion

This is the first study, to the best of our knowledge, to examine the presence, abundance and seasonality of *Ae. albopictus* along an altitudinal gradient in Italy. *Aedes albopictus* first arrived in Italy 30 years ago, and 12 years have passed since the last update of its EL there, which was then considered to be 600 m asl [12] and, until now, no thorough investigation has been performed to confirm or extend this upper limit. We document here the establishment of this species beyond its known altitudinal limit, and provide evidence that the Asian tiger mosquito can thrive in temperate regions at high altitude, even in tiny mountain villages.

The highest established *Ae. albopictus* population was observed at ~ 900 m asl (Capranica Prenestina), where its activity period exceeded 3 months. Although winter temperatures represent a relevant constraint for the range expansion of the Asia tiger mosquito, in Capranica Prenestina the mean temperature of the coldest month did not drop below − 5 °C [8] (weather data from the 3bmeteo archive at https://www.3bmeteo.com/). In accordance with this result, the species altitudinal limit in central Italy can be updated, from 600 to 900 m asl. Although this species has already been recorded in subtropical and tropical areas at altitudes above 1200 m asl [58–60], only recently has it been proved to thrive above 1000 m asl in a southern European country, i.e. Albania [18]. Our evidence, together with this latter finding, confirms that the population of *Ae. albopictus* in Italy has a higher ecophysiological plasticity [61] and more explosive spread than other southern European populations within the same genetic cluster [62, 63].

Intensive growth of the transport sector, both on a global and regional scale, and milder winter temperatures due to climate change are concomitant factors driving the range expansion of arthropod vectors [64, 65]. According to the exponential decay regression, the predicted EL of this species is ~ 1015 m asl; however, OTs located at higher elevations were also found to be positive (Guadagnolo ~ 1190 m asl). Nevertheless, with just two positive OTs found during two consecutive surveys, it is not possible to consider the population in Guadagnolo as an established one, as at the altitude of this village, the time interval of optimal environmental conditions for *Ae. albopictus* might be too short to allow stable colonization. These findings could be the result of occasional introductions that occurred during the warm season and presumably ended with the arrival of the cold season. More studies are needed to investigate this further.

The demonstrated expansion of the geographical distribution of the Asian tiger mosquito poses a serious problem for public health agencies, as it implies an increase in areas potentially exposed to *Aedes*-borne viruses and thus the necessity to earmark further resources for *Ae. albopictus* control, despite the fact that existing programmes are already under strain due to the current distribution of this vector [64, 66] (Fig. 2a). When the previously recorded altitudinal limit of *Ae. albopictus* distribution and the mean altitude of the Lazio region's municipalities were taken into account, it was considered that surveillance of this vector should be carried out in 281 municipalities (out of a total of 378, i.e. 74%), covering an area of 13,249.79 km^2^ (~ 77% of the region). However, according to our results, the surveillance area should be extended by ~ 14% and cover all the municipalities with a mean altitude up to the species’ EL (1015 m asl), i.e. nearly 94% of municipalities of the region, in which 99.5% of its inhabitants live.

Whilst the widespread presence of *Ae. albopictus* suggests that surveillance activities should be expanded, the results for the phenological metrics indicate that control efforts should be harmonized according to the changes in the abundance and days of activity of this species along the altitudinal gradient examined. Indeed, besides the impracticability of expanding the surveillance activities undertaken by public health agencies, it is not necessary to provide and maintain uniform field sampling and mosquito-control efforts among altitudinal ranges. Instead, balancing the earmarked resources according to the phenological metrics of the species is key to a successful public health campaign. For instance, the number of OTs, personnel and months of surveillance could be reduced for high altitudes without adversely affecting the effectiveness of surveillance, considering that *Ae. albopictus* abundance and days of activity were found to decrease, respectively, by 95% and 34% from the lowest to the highest altitudinal range. Global trends of increasing urbanization and a higher human footprint have a positive effect on *Ae. albopictus* abundance [19, 67]. Nevertheless, according to our results, even small human settlements consisting of a few houses and with a low population density are suitable for the Asian tiger mosquito. Indeed, the highest locality where *Ae. albopictus* was observed, Guadagnolo, is a small village with 56 inhabitants, with a population density of ~ 6 inhabitants/km^2^. Even if, on the one hand, it can be argued that population density decreases at increasing altitude [40], on the other hand the attraction of tourists to mountain villages, especially in the summer months, cannot be underestimated. It is indeed expected, under a scenario of climate change, that mountainous areas will attract more tourists in the future [68]. Hence, even tiny villages in mountainous areas of temperate regions, once considered mosquito-free, could potentially be affected by *Aedes*-borne virus outbreaks. However, it is presumed that outbreaks at high altitudes will be characterised by reduced incidence and a shorter time span due to a lower mosquito survival rate and longer extrinsic incubation period, which are factors that negatively affect vectorial capacity [69]. Nevertheless, as indicated by previous studies, the altitudinal range of some *Aedes*-borne arboviruses has extended with the spread of vectors due to climate change [70]. This is also a possibility that cannot be ruled out for central Italy, where high summer temperatures have been recorded even at elevated altitudes: for instance, the maximum recorded temperatures (August 2021) in one of our highest sampling localities (Capranica Prenestina ~ 900 m asl) remained above 30 °C for a week (weather data from the archive of https://www.3bmeteo.com/).

In conclusion, the observed 300-m shift in the EL of *Ae. albopictus* (~ 400 m predicted EL, and ~ 500 m EL when taking into consideration the positive OTs in Guadagnolo), besides allowing us to gain a more comprehensive understanding of the relationship between the distribution, abundance and phenology of this species with altitude, can also be considered a warning sign for public health agencies tasked with the surveillance of this vector in Italy and other southern European countries.

## Data Availability

The data that support the findings of this study are available from the corresponding author, FR, upon reasonable request.
